# Ex-chitin-g news on drug-induced fungal epitope unmasking

**DOI:** 10.1128/mbio.01387-23

**Published:** 2023-10-03

**Authors:** Robert T. Wheeler

**Affiliations:** 1 Department of Molecular and Biomedical Sciences, University of Maine, Orono, Maine, USA; 2 Graduate School of Biomedical Science and Engineering, University of Maine, Orono, Maine, USA; Duke University, Durham, North Carolina, USA

**Keywords:** *Candida*, antifungal agents, cell wall, chitin, calcineurin, mitogen-activated protein kinases

## Abstract

The microbial cell wall is an essential cellular organelle commonly targeted by antimicrobials. It is also a battleground of innate immune recognition where microbes can evade immune recognition by masking essential cell wall components. A recent study (A. S. Wagner, S. W. Lumsdaine, M. M. Mangrum, and T. B. Reynolds, mBio https://doi.org/10.1128/mbio.00074-23, 2023) provides insight into how echinocandin antifungals cause exposure of proinflammatory β(1,3)-glucan by driving excess chitin production in the weakened cell wall. Although many environmental and biological activities perturb cell wall integrity and regulate β(1,3)-glucan exposure, we still know little about which intracellular signaling components regulate the cell wall changes that result in disrupted cell wall architecture. Wagner et al. showed that calcineurin and the Mkc1p kinase regulate chitin deposition and β(1,3)-glucan unmasking. They further identified chitin synthesis as a key driving force in cell wall structure disruption leading to epitope exposure. Their findings highlight how fungal cell wall dynamics have important implications for antifungal immunity and future drug development.

## COMMENTARY

In the last 20 years, the fungal pathogenesis community has taken a rich foundation of basic fungal cell wall science and leveraged it to understand how it applies to host-pathogen interaction and antifungal treatment. This work by a number of laboratories has exposed the need to look at the cell wall not as a jumble of pathogen-associated molecular patterns ready for immune recognition, but rather as a dynamic structure with the capacity to deny innate immune receptors access to fungal-specific molecules (1 – 3).

Fungi build a layered cell wall structure that responds dynamically to antifungal treatment, immune attack, and environmental conditions. These responses can drive increased inflammation through increased epitope exposure, can create a cell wall that is more resistant to subsequent antifungal treatment, and can limit pathogenesis in systemic and gastrointestinal models of disease (4 – 7). Unfortunately, as our awareness of the power of fungi to regulate their cell walls has increased, this has not yet been met by a concomitant expansion in our understanding of the mechanisms that underlie their ability to mask and unmask epitopes at the cell surface. Thus, recent work by Wagner et al. (8) marks an important step forward in our understanding of the process of cell wall restructuring in the most common human fungal pathogen, *Candida albicans*.

In the past several years, the Reynolds group has sought to understand how antifungal drug treatment leads to changes in the cell wall. This has synergized with previous work from several laboratories, studying several fungal species, that has shown the echinocandin class of antifungals, which target β(1,3)-glucan synthase, reduce the total amount of cellular β(1,3)-glucan but cause exposure of β(1,3)-glucan epitopes on the fungal cell surface. This occurs both upon treatment *in vitro* and with therapy during murine infection but is not seen with other antifungal drugs such as fluconazole. Importantly, the unmasking of β(1,3)-glucan by echinocandins leads to enhanced recognition by the Dectin-1 β(1,3)-glucan receptor, inducing pro-inflammatory immune responses (1, 3).

This elegant new work by Wagner et al. (8) adds key elements to our understanding about how inhibition of β(1,3)-glucan synthesis leads to epitope exposure. They first probed the relationship between chitin synthesis and β(1,3)-glucan exposure, which has been found to correlate in other situations. Remarkably, they found that inhibition of all chitin synthesis with the antifungal drug nikkomycin Z inhibits acute β(1,3)-glucan exposure caused by caspofungin treatment. This fits with previous evidence that Chs3p, the major chitin synthase, is required for β(1,3)-glucan unmasking in *C. albicans* hyphae in areas attacked by neutrophils and that the enzyme is found at these sites (9). Similarly, Wagner et al. found that Chs3p localizes to sites of new cell wall addition in daughter cells during acute echinocandin challenge. Then, they built on their previous work that identified calcineurin, a highly conserved regulator of stress responses, as a mediator of β(1,3)-glucan exposure during caspofungin treatment (10). Specifically, they found that calcineurin works together with the mitogen-activated protein kinase (MAPK) Mkc1p to stimulate both chitin deposition and β(1,3)-glucan exposure downstream of antifungal treatment. Intriguingly, deactivation of either calcineurin or Mkc1p prevents chitin deposition and β(1,3)-glucan unmasking in about 50% of cells, and it is not clear what causes these divergent responses. Nevertheless, measurement of both chitin and β(1,3)-glucan exposure during this differential response further confirms the tight link between chitin deposition and unmasking, as the cells with greater chitin levels have more unmasking.

**Fig 1:**
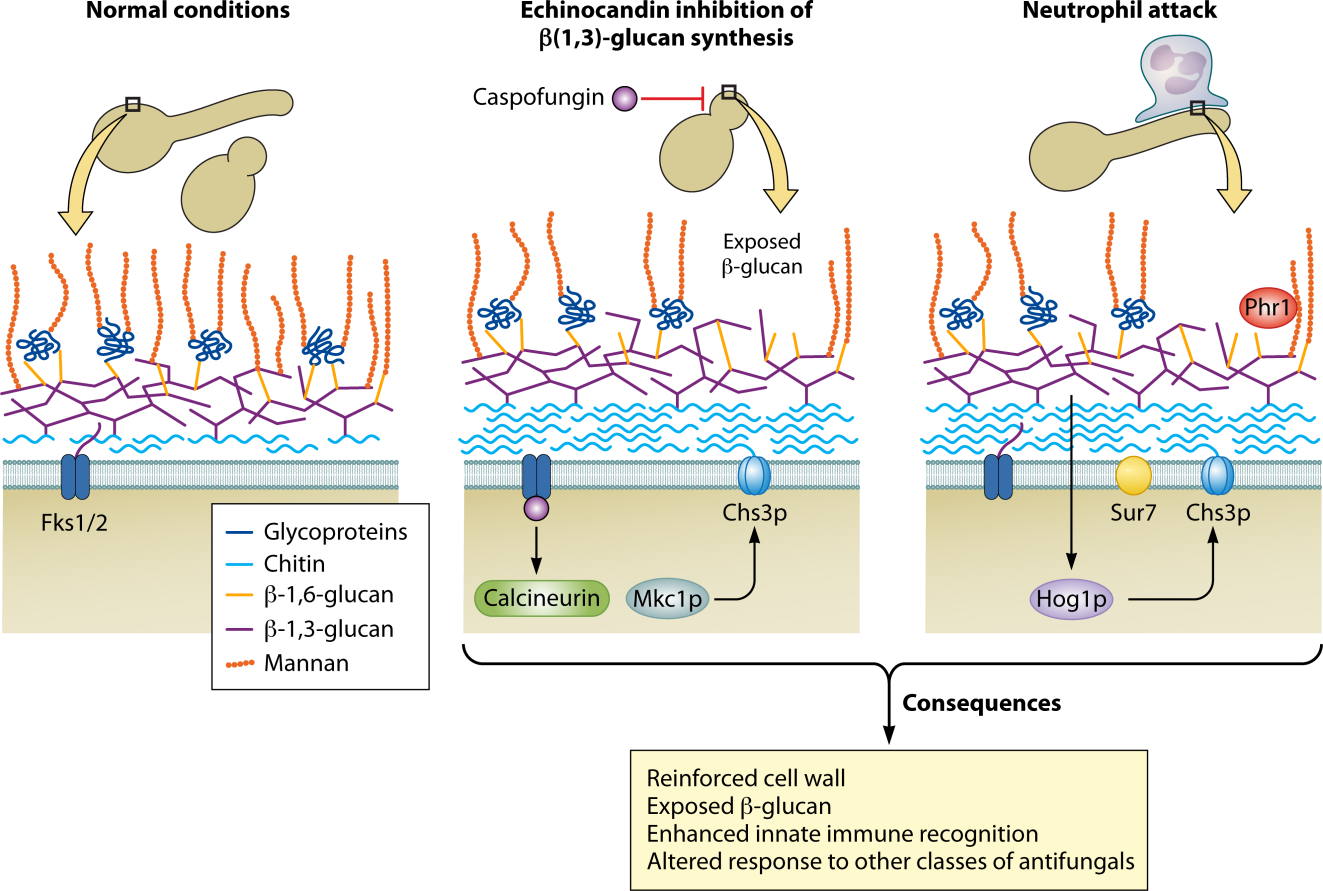
Chitin synthesis drives proinflammatory β(1,3)-glucan unmasking. Layering in the cell wall normally masks β(1,3)-glucan from recognition. Environmental (caspofungin; center) and biological (neutrophil attack; right) insults weaken the cell wall, and this triggers cell wall integrity responses that reinforce the wall. (Middle). Echinocandins trigger the activation of calcineurin and Mkc1p, which stimulates Chs3p chitin synthase activity at the perturbed cell wall. This in turn disrupts cell wall architecture which can lead to exposed β(1,3)-glucan. (Right) Neutrophil attack causes similar effects through the Hog1p kinase (bottom; consequences). These changes to the cell wall in response to insults strengthen it but enhance innate immune recognition and can alter subsequent drug susceptibility.

Taken together with previous work on immune-mediated remodeling of mature hyphal cell wall, this new work by Wagner et al. leads to a more sophisticated view of how localized perturbation of cell wall integrity acts through a network of signaling proteins (calcineurin, Mkc1p, and Hog1p) to relocalize cell wall remodeling machinery (e.g., chitin synthase Chs3p and β(1,3)-glucan transglycosylase Phr1p) to the site of weakness. Chs3p then synthesizes chitin and thereby reinforces the cell wall (Fig. 1). However, this chitin deposition leads—through unknown mechanisms—to a stronger but less intricately layered cell wall in these spots, in which some β(1,3)-glucan lies exposed on the outer surface, such that it can be more efficiently recognized by Dectin-1 and elicits stronger immune responses (1, 3).

As with all important advances, this work brings up several questions. First, what are the other signaling components besides calcineurin and Mkc1p, and how do they all interact with known signaling networks that include other MAPKs, MAPKKs (MAPK kinases), and downstream transcription factors (1)? Second, how and why does chitin deposition enhance β(1,3)-glucan exposure? Third, do echinocandins invoke the same pathways in susceptible fungi (pathogenic or not) during prophylaxis and treatment in patients?

What is the larger implication of this work? The work by Wagner et al. raises important questions relative to host-pathogen interaction, the nuts and bolts of remodeling the cell wall to reinforce it, the consequences of antifungal treatment on immune responses, and the interplay among diverse signaling molecules that regulate cell wall integrity. Perhaps the most important gap in our knowledge is how chitin synthesis and deposition in weak cell wall changes the structure of the cell wall, which strengthens it but also causes a loss of organization and exposure of normally masked epitopes. New work to 3D-model the different molecules within the organized cell wall, in conjunction with new probes to identify specific structures, will help in this cause (2).

Second, understanding the interplay between chitin- and β(1,3)-glucan-targeting drugs is crucial for developing antifungal treatment strategies that take advantage of drug-drug synergies. One pitfall to avoid is the issue that inhibiting chitin synthesis drives up chitin deposition and this makes *Candida* more resistant to echinocandin treatment (4, 7). Delineating the activities of signaling proteins such as calcineurin, Mkc1, and Hog1 may point to new strategies for combination therapy without the negative effects of compensatory changes.

Third, given that limiting chitin synthesis limits remodeling and β(1,3)-glucan exposure in response to both echinocandins and neutrophil attack, it is interesting to speculate that chitin synthesis-targeting drugs might limit the potential for increased inflammation downstream of either insult. This would have positive implications for limiting the negative effects of immunopathology (11) but could limit the ability of innate immune cells to get activated and contain fungal cells.

The elucidation of cell wall integrity signaling networks in *C. albicans* may be useful for studying the phenomenon in other fungal pathogens since there is good conservation of calcineurin and MAPKs, as well as conservation of chitin synthases, β(1,3)-glucan synthases, and β(1,3)-glucan remodeling enzymes. Furthermore, the conservation of the structural polysaccharide β(1,3)-glucan, and the use of layered cell wall structures by other fungi to control epitope availability, suggests that these findings in *C. albicans* are likely to have relevance for other fungal pathogens. In broader terms, this work toward an understanding of not just which PAMPs (pathogen-associated molecular patterns) are present, but which PAMPs are exposed to innate immune receptors, highlight the need to understand cell wall structure and dynamics in all microbes that interact with the immune system.
